# Podocalyxin Regulates Murine Lung Vascular Permeability by Altering Endothelial Cell Adhesion

**DOI:** 10.1371/journal.pone.0108881

**Published:** 2014-10-10

**Authors:** Erin J. Debruin, Michael R. Hughes, Christina Sina, Alex Liu, Jessica Cait, Zhiqi Jian, Martin Lopez, Bernard Lo, Thomas Abraham, Kelly M. McNagny

**Affiliations:** 1 The Biomedical Research Centre, University of British Columbia, Vancouver, BC, Canada; 2 UBC James Hogg Research Centre, Institute for Heart + Lung Health, Vancouver, BC, Canada; 3 Penn State College of Medicine, Penn State University, Hershey, Pennsylvania, United States of America; Vanderbilt University Medical Center, United States of America

## Abstract

Despite the widespread use of CD34-family sialomucins (CD34, podocalyxin and endoglycan) as vascular endothelial cell markers, there is remarkably little known of their vascular function. Podocalyxin (gene name *Podxl*), in particular, has been difficult to study in adult vasculature as germ-line deletion of podocalyxin in mice leads to kidney malformations and perinatal death. We generated mice that conditionally delete podocalyxin in vascular endothelial cells (*Podxl* ^ΔEC^ mice) to study the homeostatic role of podocalyxin in adult mouse vessels. Although *Podxl* ^ΔEC^ adult mice are viable, their lungs display increased lung volume and changes to the matrix composition. Intriguingly, this was associated with increased basal and inflammation-induced pulmonary vascular permeability. To further investigate the etiology of these defects, we isolated mouse pulmonary endothelial cells. *Podxl* ^ΔEC^ endothelial cells display mildly enhanced static adhesion to fibronectin but spread normally when plated on fibronectin-coated transwells. In contrast, *Podxl* ^ΔEC^ endothelial cells exhibit a severely impaired ability to spread on laminin and, to a lesser extent, collagen I coated transwells. The data suggest that, in endothelial cells, podocalyxin plays a previously unrecognized role in maintaining vascular integrity, likely through orchestrating interactions with extracellular matrix components and basement membranes, and that this influences downstream epithelial architecture.

## Introduction

Lung development is a highly regulated process requiring tight coordination between airway morphogenesis/alveolarization and vasculogenesis/angiogenesis [1], [2]. Lung alveolar function also requires the development of a tight alveolar-capillary complex to facilitate pulmonary gas exchange. The maintenance of vessel patency by cell-cell and cell-matrix interactions is a critical factor in regulating both vascular permeability and proper airway function. Disruption of the pulmonary capillary barrier, which occurs frequently during acute lung inflammation, causes leakage of fluid, plasma constituents and cells into interstitial tissues and airspaces and thereby contributes to increased morbidity and mortality of patients with acute lung injury [3], [4]. In addition, abnormal vessel growth and vascular dysfunction contribute to a number of pediatric and adult lung pathologies, including bronchopulmonary dysplasia and chronic obstructive pulmonary disease [5], [6]. In experimental models, the loss of genes regulating vessel development and patency or the administration of anti-angiogenic drugs can lead to defects in alveolarization and lung structure [7]–[9]. Thus, lung vessel patency and the regulation of permeability are closely linked to lung structure and function.

Podocalyxin (gene name *Podxl* and also known as podocalyxin-like protein 1 (PCLP1)) is a cell surface sialomucin that shares genomic and structural similarities to CD34 and endoglycan [10], and was initially identified as a heavily sialylated protein of kidney glomerular epithelial cell glycocalyx [11]. Podocalyxin is also expressed by vascular endothelia [12]; hematopoietic stem cells [13]–[15]; hemangioblasts [16]; stress erythroid progenitors [17]; mesothelial cells lining many organs [18], neurons [19] and metastatic cancers [20]. Functionally, although podocalyxin can act as an adhesive receptor on rare high endothelial venules (HEV), under most circumstances we and others have found that podocalyxin acts as an anti-adhesin through its negatively-charged mucin domain and this has been shown to play an important role in cell migration, adhesion and lumen formation [10], [21]. Likewise, we have shown that high levels of podocalyxin expression result in apical domain expansion, driving adherens and tight junctions toward the basolateral surface and altering integrin localization [18], [22].

Gene deletion studies have shown that both podocalyxin and CD34 play an important role in the development and function of blood vessels. *Cd34^-/-^* mice are more susceptible to autoimmune arthritis as a result of increased vascular permeability at the earliest stages of disease [23]. Additionally, in tumor-angiogenesis models, loss of CD34 results in altered vessel structure and vascular integrity but normal vessel density within developing tumors [24]. Likewise, during early embryogenesis, Strilic *et al.* determined that podocalyxin and CD34 provide an anti-adhesive function in formation of nascent lumens between endothelial cords and that the loss of podocalyxin was sufficient to delay opening of the aortic vascular lumen [21]. Interestingly, *Podxl^-/-^* mice have otherwise normal vascular beds within most tissues just prior to birth [18]. Unfortunately, conventional, germ-line deletion of the *Podxl* gene leads to developmental malformations and perinatal lethality, which preclude analyses to understand the importance of podocalyxin in adult vasculature.

To examine the function of podocalyxin in adult vessels, we generated mice with a floxed podocalyxin allele (*Podxl^F/F^*) and then bred this strain with mice expressing the Cre recombinase under the control of a VE-cadherin promoter (*Cdh5-Cre*) thereby deleting podocalyxin selectively in vascular endothelia (*Podxl* ^ΔEC^ strain) [25]. We found that loss of podocalyxin from the lung vasculature results in changes to the lung structure and function including increased lung volume when inflated under constant pressure (25 cm H_2_O) and changes to the matrix composition. In addition, the loss of podocalyxin results in a striking increase in pulmonary vascular permeability, particularly during acute inflammation. While we observe no changes in expression of classic endothelial cell markers, podocalyxin-deficient endothelial cells are unable to spread efficiently on laminin but exhibit enhanced adhesion to fibronectin. Furthermore, *Podxl* ^ΔEC^ endothelial cells upregulate transcripts for integrins that are responsible for binding to extracellular matrix proteins. In summary, our data reveals a key role for post-natal podocalyxin expression in the cell-matrix interactions that regulate vascular permeability in the lung.

## Materials and Methods

### Animals

All experiments and procedures were performed in accordance with the requirements of the Canadian Council on Animal Care (CCAC) and the UBC Animal Care Committee approved all animal experimental protocols (protocol #A06-1483). Mice were maintained in a specific pathogen-free facility at the Biomedical Research Centre (Vancouver, BC, Canada). *Cdh5*-Cre mice (B6.Cg-Tg^(Cdh5-cre)7Mlia^/J) were purchased from the Jackson Laboratory (Bar Harbor, ME). Conditional podocalyxin knockout mice were generated and crossed with *Cdh5-Cre* mice to generate mice carrying endothelial-specific deletion of podocalyxin. Male and female mice were used for all experiments unless otherwise stated.

### Generation and genotyping *Podxl* ^ΔEC^ mice

**Figure 1:**
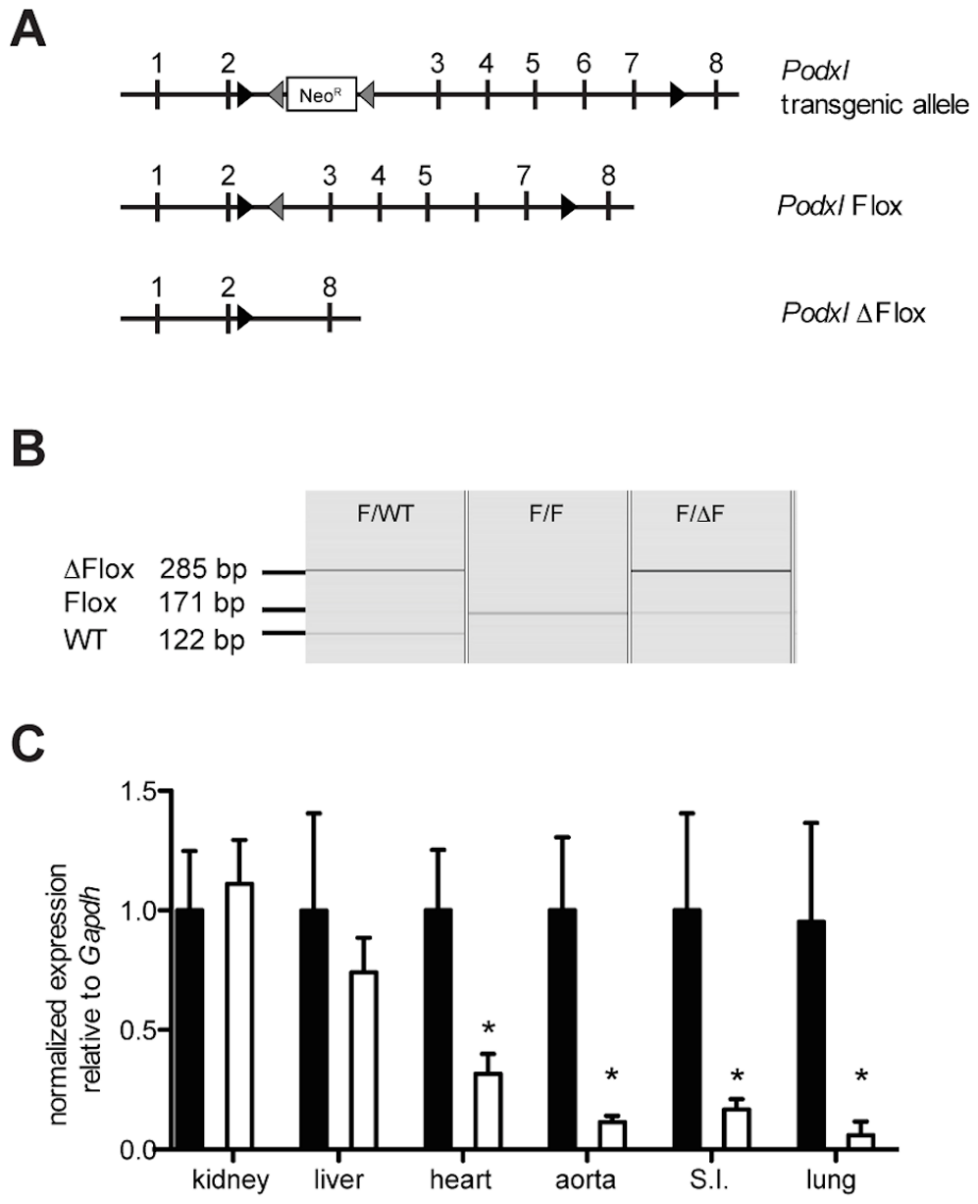
Conditional deletion of the *Podxl* locus in endothelial cells. (**A**) Schematic representation of the *Podxl* transgenic allele, floxed allele (Podxl^F/F^) and deleted allele (*Podxl* ^Δ*F/F*^). Exons are depicted as vertical lines. Inserted loxP and frt sites are depicted with black and grey arrowheads, respectively. The Neo^R^ cassette is represented by a box. (**B**) Capillary gel electrophoresis of genomic DNA isolated and amplified (PCR) from primary lung endothelial cells prepared from mice harboring wild type (WT, 122 bp), floxed (Flox, 171 bp) or functionally deleted (via *Cdh5*-Cre) *Podxl* alleles (ΔFlox, 285 bp) (**C**) qRT-PCR evaluation of podocalyxin mRNA in highly vascularized adult tissues harvested from *Podxl* ^F/F^ (black bars) and *Podxl* ^ΔEC^ (white bars) mice (n = 3–6). Expression levels were quantified relative to *Gapdh* and then normalized to the mean *Podxl* expression in the *Podxl^F/F^* tissues. *Significantly different compared to *Podxl^F/F^* mouse tissue where P<0.05 by Student's *t* test. Error bars represent the SEM.

To generate mice that conditionally delete podocalyxin, a mutant *Podxl* genomic targeting construct introduced a neomycin resistance cassette (Neo^R^) between exons 2 and 3 flanked with frt sites along with two loxP recombination sequences upstream of exons 3 and 8 (**Fig. 1A**). R1 ES cell clones carrying the mutant vector were injected into albino C57Bl/6J-Tyr-C2J blastocysts. Mice exhibiting high chimerism were crossed to C57Bl/6J mice and offspring bearing a germ line mutation of the *Podxl* allele were identified by PCR. The neomycin cassette was removed *in vivo* by breeding these mice to mice ubiquitously expressing Flp-recombinase [26]. Endothelial-specific Cre recombinase-mediated excision of exons 3–7 was achieved by crossing the resulting floxed *Podxl* mice with mice expressing Cre under control of the *Cdh5* promoter [25].

For the purposes of genotyping mice, genomic DNA was isolated from ear clips or isolated cell pellets by proteinase K digestion and ethanol precipitation as described previously [18]. The diagnostic PCR strategy to identify successful integration of the Neo^R^ gene between frt sites in intron 2 used primers that bound upstream of the first loxP site (5′-TGGCCTCAAACTCAGAGAT CTATC-3′), within NeoR cassette (5′-CTTCTATGAAAGGTTGGGCTT-3′), and downstream of the final frt sites (5′-ACCGGTGAAGTTCCTATACT-3′) resulting in a 437 bp product to indicate germ line transmission. Flp-dependent deletion of the Neo^R^ cassette was confirmed using primers upstream of the first loxP site (5′-CTCATACTACCAGTCAGTTGG-3′) and downstream of the final frt site, which results in a diagnostic 632 bp product in deleted mice, or a 519 bp band in mice retaining the Neo^R^ cassette. Successful deletion of *Podxl* ^F/F^ exons 3–7 (*Cdh5-Cre* mediated) was evaluated in isolated lung endothelial cells using primers upstream of the first loxP site, upstream of the final loxP site (5′-TTACTCTAGGTAGCCCAGTT-3′) and downstream of the final loxP site (5′-TCTCCAGCGTTAGAGACAAG-3′). These result in a 285 bp product in deleted cells and a 171 bp product in non-deleted cells.

### Histology and Morphometric Analysis

Experimental mice were anesthetized with ketamine/xylazine. For constant volume inflation, the lungs were inflated with 0.8 mL of 1% low melting agarose (Invitrogen) (closed chest cavity) and the thoracic cavity was thereafter dissected and lungs were excised after the agarose had cooled. For constant pressure inflation, the chest cavity was opened and the lungs were inflated with agarose to a pressure equal to 25 cm water. After lung inflation, the volume of agarose-inflated lungs were measured by water displacement [27]. Tissues were post-fixed for 24 h in 10% buffered formalin before processing. Mean linear intercept (MLI) was determined from 4 µm lung histological sections stained with hematoxylin and eosin. Images were taken of 8 randomly chosen fields and the MLI was calculated with a grid of 130 lines and 250 points and a line length of 118.93 µm (ImagePro, MediaCybernetics, Bethesda, MD) [28].

All other tissues harvested from PBS-perfused mice were post-fixed for 24 h in 4% paraformaldehyde and transferred to 70% ethanol prior to processing for paraffin embedding. To assess podocalyxin expression in tissues, 4 µm deparaffinized sections underwent heat-mediated citrate antigen retrieval and were incubated with goat anti-podocalyxin antibody (R&D Systems) or isotype control overnight. Antibody binding was detected using VECTASTAIN ABC (Goat IgG) kit (Vector Labs), developed with DAB (3,3′-diaminobenzidine) and counterstained with Methyl Green or Hematoxylin (Vector Labs). To quantify elastin density, lung sections were stained with Gomori's aldehyde fuchsin and images were taken of 8 randomly chosen parenchymal fields. Elastin density was calculated by thresholding the area of stain to the total tissue area using ImageJ (NIH) [29]. To quantify vessel density, images were taken of 5 randomly chosen sites, which did not include conducting airways, from 4 µm histological sections of formalin-fixed, paraffin-embedded lung tissue stained with vWF (Sigma). Vessel density was calculated with a grid of 192 points to determine the ratio of the number of vWF immunoreactive points to the number of total lung parenchyma points using ImageJ (NIH) [30].

To visualize junctional proteins, paraffin-embedded lung sections underwent heat-mediated antigen retrieval and were incubated with mouse anti-ZO-1 (Invitrogen) and rabbit anti-claudin 5 (Abcam) overnight. Donkey anti-mouse AlexaFluor 555 and donkey anti-rabbit 647 (Invitrogen) were used to detect antibody staining and nuclei were labeled with DAPI. Fluorescent images were acquired with the Leica AOBS SP2 laser scanning confocal microscope (Leica, Heidelberg, Germany) running Zeiss LSM 510 software (Carl Zeiss, Toronto, ON, Canada). Images were processed using ImageJ.

### Determination of vascular permeability and edema

Vascular permeability was quantified by a modified Miles assay as previously described [31]. Female mice were anesthetized with isoflurane before intratracheal instillation of 2 mg/kg LPS (Sigma) 24 h before sacrifice. Naïve or LPS treated mice were injected intravenously (i.v.) with 20 mg/kg Evans Blue Dye (EBD) 1 h before sacrifice. Mice were anesthetized with an intraperitoneal injection of ketamine and xylazine (450 µl of 10 mg/mL ketamine/1 mg/mL xylazine solution) and then perfused with PBS. The lung tissue was excised and weighed. EBD remaining in the tissue parenchyma was extracted in formamide for 72 h at 37°C. The optical density (OD) of the supernatants was read at 620 nm and 740 nm on a spectrophotometer, and the amount of dye per mass of tissue (µg dye/g tissue) was calculated after a tissue-specific correction factor was applied [31]. Lung edema was measured in naïve mice. Lungs were excised and weighed immediately to determine wet weight. Dry weight was determined after lungs were dried for 36 h at 60°C. Lung edema was presented as a ratio of wet lung weight to dry lung weight.

### Lung function measurements

Mice were anesthetized with an intraperitoneal injection of ketamine and xylazine (450 µl of 10 mg/mL ketamine/1 mg/mL xylazine solution). Mice were tracheotomized and intubated with an 18-G catheter and all parameters were measured with a flexiVent apparatus (SCIREQ,Montreal, QC). Mice were given 0.8 mg/kg pancuronium bromide to prevent spontaneous breathing. Respiratory frequency was set at 160 breaths/min with a tidal volume of 0.2 mL, and a positive end-expiratory pressure of 2 to 4 mL H_2_O was applied. A “snapshot perturbation” maneuver was imposed to measure resistance (R), compliance (C), and elastance (E) of the whole respiratory system (airways, lung, and chest wall). Forced oscillation perturbation (“primewave-8”) was consequently applied, and resulted in airway resistance (Rn), inertia of the air, tissue damping (resistance) (G), tissue elasticity (H), and tissue hysteresivity (tissue damping [G]/H). Maximal pressure-volume loops (PV-loops) were subsequently determined to measure vital (total) lung capacity, IC from zero pressure (B), form of deflating PV loop (K), static C (Cst), static elastance (Est), and hysteresis (area between inflating and deflating part of the loop). For each parameter, an average of three measurements was calculated and depicted per mouse [32].

### Multiphoton and second harmonic generation imaging

The laser used for second harmonic generation (SHG) as well as the multi-photon excitation fluorescent microscopy (MPEF) was a mode-locked femto-second Ti:Sapphire Tsunami (Spectra-Physics, Mountain View, CA) synchronously pumped by a Millenia Xs J (Spectra-Physics) diode-pumped solid-state laser capable of delivering up to 10 W pumping power at 532 nm. The laser was directed to a Leica AOBS scan head coupled with Leica upright microscope system (Heidelberg, Germany) at an average power that was below the damage threshold of the samples. Upon entering the Leica microscope system, the laser beam was directed to the scanning mirrors, then through a 690 nm long pass dichroic mirror (690 DCXRU, Chroma Technology) and subsequently focused on the specimens through a 20x/0.95 NA water dipping objective lens (Olympus) and the backscattered emission from the sample was collected through the same objective lens. Leica Confocal Software TCS SP2 was used for the image acquisition. Non-de-scanned detectors in the reflection geometry located close to the objective were used for capturing the 3D images. In the non-de-scanned PMT detectors (R6357, Hamamatsu, Shizuoka, Japan), a 700 nm short pass filter (E700SP, Chroma Technology, USA) was used to prevent the scattered IR laser radiation from reaching the detector and a 455 long pass dichroic beam splitter (455 DCXRU, Chroma Technology, USA) was used to separate SHG signal from the MPEF signal.

For 3D image data set acquisition, the multiphoton excitation beam was first focused at the maximum signal intensity focal position within the tissue sample and the appropriate PMT levels (both the gain and offset levels) were then selected to obtain the pixel intensities within range of 0–255 (8-bit images) using a color gradient function. Afterward, the beginning and end of the 3D stack (i.e., the top and the bottom optical sections) were set based on the signal level degradation. Series of 2D images for a selected 3D stack volume were then acquired at slow scan speed i.e., 10 s per 512×512 pixels. The 3D stack images with optical section thickness (z-axis) of approximately 1.12 µm were captured from tissue volumes. For each tissue volume reported here, z-section images were compiled and finally the 3D image restoration was performed using Volocity software (Improvisions, UK). A noise-removal filter, whose kernel size of 3×3, was applied to these 3D image volumes.

### Reagents

Matrix used for the adhesion assays included fibronectin (Millipore), collagen type I (Serva Electrophoresis), laminin (BD BioSciences), and 0.2% gelatin (Sigma). EC Media is comprised of DMEM with 1x penicillin/streptomycin, 1x non-essential amino acids, 1 mM sodium pyruvate and 2 mM L-glutamine, 20% FBS, 100 ng/mL endothelial cell growth supplement (BD Biosciences), 25 mM HEPES and 90 µg/mL heparin sulfate.

### Flow cytometry

Primary mouse endothelial cells (mECs) were harvested from culture plates using TrypLE Express and stained with one or more of the following fluorochrome conjugated antibodies: Podocalyxin-APC (R&D Systems), CD31-PeCy7 (clone 390 eBiosciences), CD45-PerCP (clone 30-F11, BD Pharmingen), CD34-APC (BD Biosciences), ICAM-2 efluor405 (clone 3C4 ebiosciences), Tie-2 PE (BD Biosciences) and endoglin PE (clone MJ7/18, ebiosciences), α4 integrin-PE (R1-2, ebiosciences), α5 integrin PE (eBioHMa5-1, ebiosciences), α6 integrin eFluor405 (GoH3, ebiosciences), αV integrin (RMV-7, ebiosciences), β1 integrin PE (HMb1-1, ebiosciences), β3 integrin (2C9.G3, ebiosciences). 10,000 events were collected for each flow cytometry sample.

### Real-time qPCR

RNA was isolated from cultured cells and tissue using RNeasy mini kits (Qiagen) and Trizol (Invitrogen), respectively. RNA quantification was performed using a ND1000 spectrophotometer (Nanodrop). Reverse transcription was performed using the High Capacity cDNA Reverse Transcription Kit (Applied Biosystems). Real time qPCR (qRT-PCR) gene expression analyses were performed using PerfeCTa SYBR Green FastMix (Quanta Biosciences) on a 7900 HT instrument (Applied Biosystems). The relative mRNA expression was normalized to a simultaneous amplification of *Gapdh* transcripts. The results are expressed as the average expression in each tissue (relative to Gapdh) and normalized to the gene expression in control *Podxl* ^F/F^ tissues (set to 1). The primers used for gene expression analysis are listed in **Table 1**.

**Table 1:** Real time qPCR primer list.

Gene Name	Forward Primer (5′-3′)	Reverse Primer (5′-3′)
*Col1a1*	CCAAGGGTAACAGCGGTGAA	CCTCGTTTTCCTTCTTCTCCG
*Col1a2*	TGTTGGCCCATCTGGTAAAGA	CAGGGAATCCGATGTTGCC
*Col4a1*	CTGGAGAAAAGGGCCAGAT	TCCTTAACTTGTGCCTGTCCA
*Col4a3*	CAAAGGCATCAGGGGAATAACT	ATCCGTTGCATCCTGGTAAAC
*Eln*	TGGTATTGGTGGCATCGG	CCTTGGCTTTGACTCCTGTG
*Fn1*	AATCCAGTCCACAGCCATTC	TAGTGGCCACCATGAGTCCT
*Gapdh*	CGTGCCGCCTGGAGAAACC	TGGAAGAGTGGGAGTTGCTGTTG
*Itga1*	GGACAGAGAAAGAAGAACAGG	CACGTTGAGGTCTTTTACAGC
*Itga2*	GGATACCCTGCCTTAAAGAGTG	GGTGGATCGGGTTAAGTGAAG
*Itga3*	GAGGATATGTGGCTTGGAGTG	ATCTCGTTGTGGTATGTCTGC
*Itga4*	TCTATCGTGACTTGTGGGCA	AGTCCAGTACGATGATCCCG
*Itga5*	AGCTGGATGTGTATGGGGAG	CAGCTCAGGCTGGAGAAGTT
*Itga6*	CTCCTAACAGAATTGACCTCCG	CTGAACTCTCGATGACAACCC
*Itga8*	GGGCATTTGGAAAAGGGAAAG	AAGGTGAAATGGGACTGGTG
*ItgaV*	ACTGGGAGCACAAGGAGAACC	CCGCTTAGTGATGAGATGGTC
*Itgb1*	GGGTATTTGTGAATGTGGTGC	TTGGTGAGATTGAAGTGGGAG
*Itgb4*	GCTTTGTGTTCCAGGTGTTTG	TGTGGGACGCTGACTTTG
*Itgb5*	ACTGCTAAGGACTGCGTTATG	GGTTTGAGGCTTTGGAACTTG
*Lama4*	AAGAAACCTTAGGAGTTGGTTATGGA	ATAAAACTTTGCCCGTTGAAATATG
*Lama5*	ACCCAAGGACCCACCTGTAG	TCATGTGTGCGTAGCCTCTC
*Lamb1*	GGCAAACTGCAAAGTCTCG	CTGGAGGTGTTCCACAGGTC
*Lamc1*	TGCCGGAGTTTGTTAATGCC	CTGGTTGTTGTAGTCGGTCAG
*Podxl*	TACACACAAACCATTGGGCA	CACGGGAGTCCCATTAGAGA

### Western Blots

Lung tissue from *Podxl^F/F^* and *Podxl* ^ΔEC^ mice (perfused with PBS) were homogenized in RIPA buffer and protein concentrations quantified by BCA (Thermo Scientific). Protein samples were diluted with 4x sample buffer, resolved on 8% SDS-PAGE gel under denaturing conditions, and transferred to PVDF membranes. Membranes were blocked with BSA and incubated overnight at 4°C with antibodies to ZO-1 (Invitrogen), claudin-5 (Abcam), VE-cadherin (gift from Volkhard Lindner), tropoelastin (Elastin Products Company) and actin (Ablab.ca). Fluorescently labeled secondary antibodies were used as appropriate and visualized on the Odyssey Imager system (LI-COR Biosciences).

### Cells

Mouse primary lung endothelial cells (mEC) were isolated from perfused lungs [33]. Lungs were minced, digested with Collagenase/Dispase (Roche Diagnostics), stained with fluorochrome-conjugated antibodies. Primary mECs (CD31^+^CD45^−^) were isolated by flow cytometry using a BD FACSAria. Cells were plated at a density of 2×10^5^ cells per well in a 24-well plate coated with 0.2% (w/v) gelatin. Primary mECs were grown in EC Media and maintained for experiments until passage five.

### Static adhesion assay

In a 96-well plate, wells were coated with 10 µg/mL fibronectin, collagen I, laminin or 0.2% gelatin and then blocked with 1% BSA. Subsequently 1×10^5^ mECs per well were plated in the coated wells and allowed to adhere for 90 min. The wells were washed with PBS. The number of adherent cells in each well was quantified by crystal violet staining as previously described [34], [35]. All conditions of the experiment were performed in triplicate.

### Spreading assay

Primary mECs (1×10^5^ cells) were seeded onto 0.4 µm 24-well cell culture inserts coated with fibronectin, laminin or collagen I (BD Biosciences) and incubated for 48 h at 37°C. Images were acquired using OpenLab 4.0 (PerkinElmer) and cell spreading was defined by % image area covered by cells using ImageJ software (NIH).

### Statistical analysis

Statistical analysis was performed using Prism 5 (GraphPad Software). Comparisons between *Podxl* ^F/F^ and *Podxl* ^ΔEC^ used the Student's *t* test. For normalized data, a one-sample *t* test was used to determine if the test value mean was significantly different than the normalized value (hypothetical value  = 1). For comparisons between multiple variables, statistics were assessed using ANOVA with a Bonferroni post-test. p<0.05 was considered as significant.

## Results

### Vascular endothelial-specific deletion of podocalyxin

**Figure 2:**
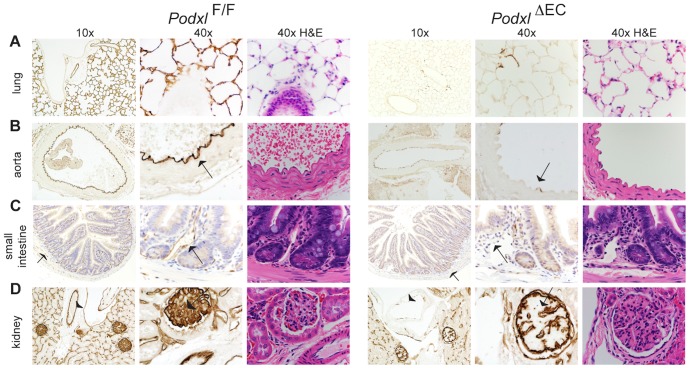
*Cdh5*-Cre drives efficient deletion of podocalyxin in the lung. (**A**) Podocalyxin expression is completely abrogated in the lung of *Podxl* ^ΔEC^ mice. (**B**, **C**) Within the aorta and vessels of the small intestine (arrows), podocalyxin is deleted in all but a few isolated cells in *Podxl* ^ΔEC^ mice. (**D**) Within the kidney, podocalyxin (brown staining) is efficiently deleted in the glomerular endothelial cells (arrows, 40x mag. panels) and larger vessels (arrowheads, 10x mag. panels). Positive staining in the glomerulus of *Podxl* ^ΔEC^ kidney are likely podocyte epithelial cells. Adjacent H&E sections included demonstrating normal morphology with loss of podocalyxin expression in these tissues.

**Figure 3:**
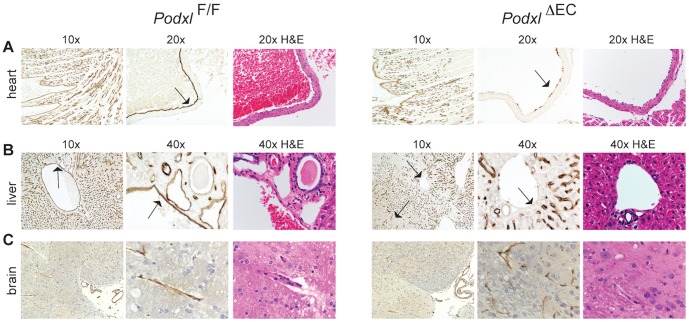
*Cdh5*-Cre fails to efficiently delete podocalyxin in a subset of organ vascular beds. (**A**) In the wildtype heart podocalyxin is expressed by all endothelial cells. In the *Podxl* ^ΔEC^ mice, podocalyxin is efficiently deleted in large vessels such as the pulmonary artery (arrow, 20x mag. panels) but deletion in the smaller trabecular vessels of the heart muscle is variable (see 10x mag. panels). (**B**) In the liver of *Podxl* ^ΔEC^ mice, podocalyxin expression is ablated in the major vessels (portal and central veins, hepatic artery) but not in sinusoidal endothelial cells. (**C**) In the brain, podocalyxin is normally expressed in the ventricles and endothelial cells, including the microvasculature. Sections of the brain from *Podxl* ^ΔEC^ mice display similar staining to control mice indicating poor recombination of loxP sites by *Cdh5*-Cre in brain. Adjacent H&E sections demonstrate normal gross morphology podocalyxin-deficient tissues.

Conventional *Podxl^-/-^* mice die shortly after birth from a glomerular podocyte defect, precluding the evaluation of its post-natal function in other tissues and cells including endothelium [18]. To circumvent this problem, we used homologous recombination in ES cells to generate a conditional “floxed” allele (*Podxl^F/F^*) to delete exons 3–7 through Cre-mediated recombination (**Fig. 1A**). The resultant mice were backcrossed >5 generations onto a C57Bl/6 background and then to *Cdh5*-Cre mice, leading to endothelial-cell specific deletion of floxed genes in major and minor vessels of most tissue beds [25]. The resulting *Podxl* ^ΔEC^ mice are viable, fertile and grossly indistinguishable from *Podxl* ^F/F^ littermates. Genotyping of purified lung endothelial cells by PCR revealed highly efficient *Podxl* deletion (**Fig. 1B**). Analysis of podocalyxin expression by qRT-PCR (**Fig. 1C**) and histology (**Fig. 2A–C**) confirmed the absence of podocalyxin in most major vascular beds including the lung, small intestine and aorta. In the kidney, where the bulk of podocalyxin expression is in the glomerular epithelial cells (podocytes), we could not detect a noticeable difference in mRNA levels (**Fig. 1C**). However, by immunohistology it is clear that podocalyxin is ablated in the endothelial cells found at the centre of the glomerulus (arrows) and in large vessels (arrow heads) (**Fig. 2D**), while the residual podocalyxin is expressed in the kidney podocytes of the glomeruli and the ductal cells. The vascular beds of a number of tissues exhibited extensive residual expression in endothelial cells including the heart, liver and brain (**Fig. 3**) and we attribute this to previously documented subtleties of the *Cdh5-Cre* deleter strain [25].

### Deletion of vascular *Podxl* contributes to changes in lung compliance and mislocalization of structural matrix proteins

**Figure 4:**
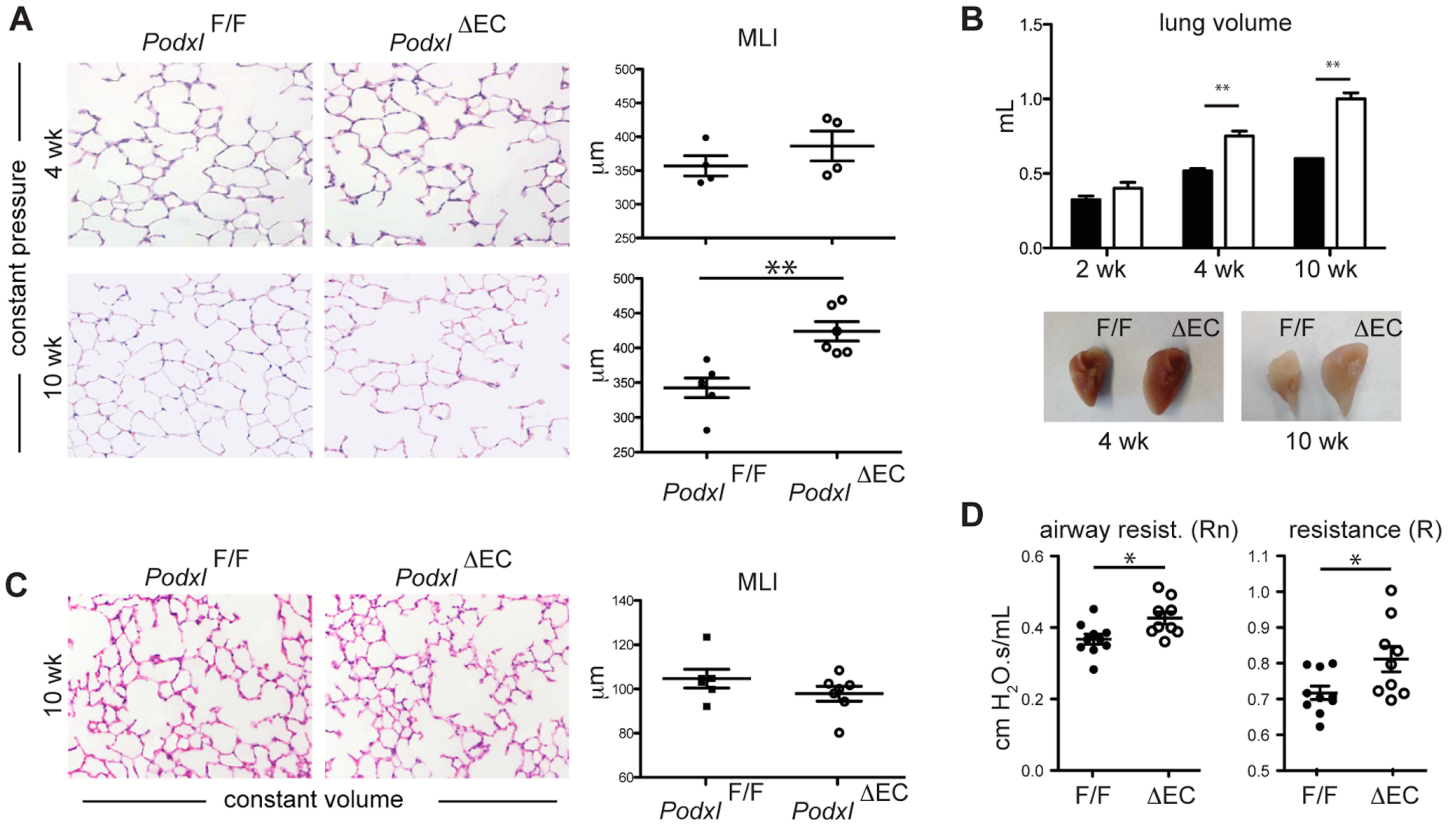
Deletion of vascular podocalyxin contributes to structural and functional changes in the lung. (**A**) H&E stained sections of lungs inflated to 25 cm H_2_O with open thoracic cavity from *Podxl^F/F^* and *Podxl* ^ΔEC^ mice obtained at 4 weeks and 10 weeks post-natal. Loss of podocalyxin results in increased mean linear intercept (MLI) at 10 weeks of age. The MLI values were determined by computer-assisted image analysis (n = 6 mice per genotype). (**B**) Mean lung volumes of *Podxl^F/F^* and *Podxl* ^ΔEC^ mice at 2, 4 and 10 weeks (n = 4–6 mice per genotype). Representative images of inflated lungs at 4 and 10 weeks are shown below the graphs. (**C**) H&E stained sections of lungs inflated with constant volume with closed chest wall from adult *Podxl^F/F^* and *Podxl* ^ΔEC^ mice (n = 6 mice per genotype). (**D**) Resistance measurements from primewave-8 (Rn) and snapshot (R) perturbation. *Significantly different with P<0.05 by Student's *t* test; **Significantly different with P<0.01 by Student's *t* test at each time point. The error bars represent the SEM.

Due to the highly efficient deletion of podocalyxin in lung vessels, we focused on this tissue to assess its role in vascular function. A number of vascular-related gene knockouts exhibit defects in lung development and maintenance of functional architecture, highlighting the importance of the vasculature in this organ [2]. Although previous analyses of neonatal *Podxl^-/-^* mice (conventional, germ-line knock-out) showed no defects in lung structure [18], much of the lung development, including extensive alveolarization, occurs post-natally. To determine whether post-natal alveolarization was altered by the loss of podocalyxin in lung vessels, we examined the architecture of the lungs of mice at different post-natal time points. Histological analyses of matched lung tissue sections (sampled from lungs inflated to a fixed pressure with an open thoracic cavity) revealed normal airspaces during alveolarization (weeks 1–3) in *Podxl* ^ΔEC^ mice but a clear increase in airspace size in older mice as measured by mean linear intercept (MLI) (**Fig. 4A**) [28]. However, the observed increase in airspace size was concordant with an increase in total lung volume (**Fig. 4B**) that may be indicative of an increase in lung compliance when inflating lungs using the open thoracic cavity method. Thus, to determine whether increased air-space size in *Podxl* ^ΔEC^ mice was due to alveolar simplification, the lungs of adult mice were inflated with a constant volume with the chest wall closed. Histological analyses of lungs under these experimental conditions revealed that wild type (*Podxl* ^F/F^) and *Podxl* ^ΔEC^ mouse lungs have comparably sized airspace with normal architecture (**Fig. 4C**). Together, these results suggest that deletion of *Podxl* in lung vasculature alters lung structural properties but does not grossly alter alveolarization.

The expansion of lung volume upon inflation at constant pressure (open thoracic cavity) suggests that *Podxl* expressed in the lung vasculature may influence lung mechanics, including compliance. To test this, we used an invasive pulmonary function apparatus (flexiVent, SCIREQ) to assess lung function in wild type and *Podxl* ^ΔEC^ mice. This approach allowed us to examine functional respiratory parameters similar to those used in human studies [32]. Although we did not detect changes in total lung capacity or compliance (data not shown), *Podxl* ^ΔEC^ mice exhibited increased total lung (R) and airway (Rn) resistance under closed-chest conditions (**Fig. 4D**). In summary, our data suggest that vascular loss of podocalyxin leads to subtle but discernable alterations in lung resistance, but not lung compliance.

**Figure 5:**
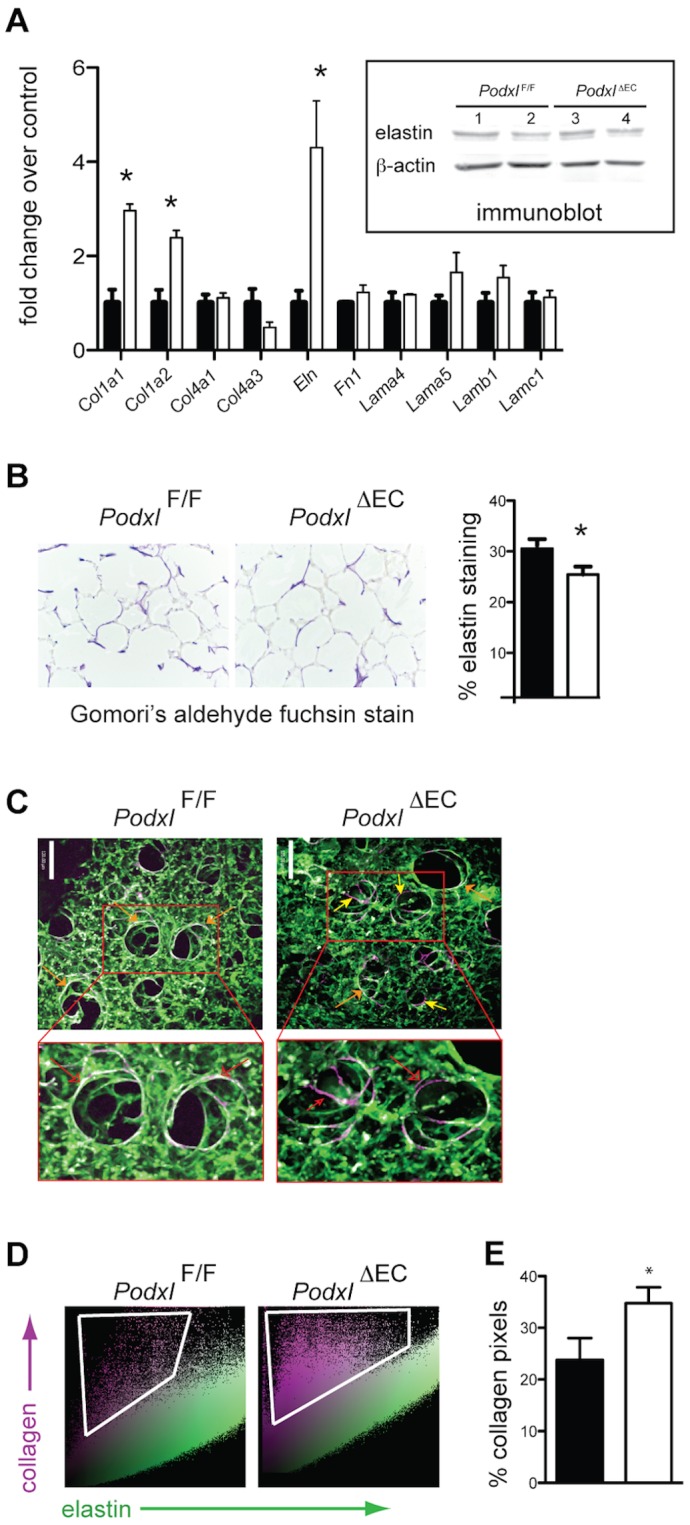
Podocalyxin deletion results in altered matrix deposition in the lung. (**A**) Whole lung tissue from *Podxl^F/F^* (black bars) and *Podxl* ^ΔEC^ (white bars) mice was assessed for expression of matrix related transcripts by qRT-PCR (n = 3 mice per group) and for tropoelastin protein by Western blot (inset). Immunoblot lanes are from whole lung tissue lysates and each lane is a sample of lung lysate prepared from a single mouse. (**B**) Representative images of sections stained with Gomori's aldehyde fuchsin stain to highlight elastin. The % elastin staining was quantified by the threshold area of elastin staining to total tissue area (n = 6 mice per genotype). (**C**) Representative optically magnified SHG image originating from the collagen matrix overlaid with the MPEF images (scale bar  = 120 µm). These are 3D extended focus views representing ∼150 µm thick tissue section. The collagen appeared to be in the form of spirally wound collagen (violet color) while the lung elastin (green color) consisted of fine fibers. Areas of fibrillar collagen co-localized with elastin appear white in colour (orange arrows). While collagen-elastin co-localization is common (e.g., see *Podxl^F/F^* lung SHG images), the *Podxl* ^ΔEC^ exhibit areas of elastin-free collagen (violet) primarily in larger alveolar spaces (yellow arrows). Images shown are representative of 2 images per mouse and 3–5 mice per group. (**D**) The representative scatter plots of the images presented in (C) where SHG pixel intensities (y-axis) are plotted as a function of elastin pixel intensities (x-axis). Elastin-free collagen SHG signals are considerably higher in *Podxl* ^ΔEC^ lungs when compared to *Podxl^F/F^* lung samples. (**E**) Quantification of violet elastin-free collagen pixels in the scatter plots represented in (D). The % elastin-free collagen is calculated base on the number of pink pixels compared to the total number of pixels in each dot plot. *Significantly different with P<0.05 by Student's *t* test when compared to *Podxl^F/F^* mice. The error bars represent the SEM.

To further delineate the cause of increased lung volume in *Podxl* ^ΔEC^ mice, we evaluated the expression of matrix related genes in whole lung tissue. We found increased transcripts for collagen I (*Col1a1* and *Col1a2*) and elastin (*Eln*), but not collagen IV (*Col4a1* and *Col4a3*) (data not shown) in *Podxl* ^ΔEC^ lungs (**Fig. 5A**). However, the amount of tropoelastin protein was comparable in *Podxl* ^ΔEC^ lungs and wild type lung tissue (**Fig. 5A** **, inset**). Collagen type 1 and elastin are essential components of the lung extracellular matrix and are required to maintain normal structure and function of the lung [36]–[39], while collagen type IV and laminin-8 (laminin α4β1γ1 are components of the endothelial basement membrane in the lung [40]. To understand whether the changes in gene transcription corresponded with a change in protein expression levels, we stained lung sections with Gomori's aldehyde fuchsin. Despite the increased levels of elastin transcripts and comparable tropoelastin protein content, we found that the density of elastin fiber staining was marginally decreased in the parenchyma of *Podxl* ^ΔEC^ lungs (**Fig. 5B**). This discrepant pattern of elastin transcript and elastin fiber content has previously been identified during the early stages of animal models of emphysema [41]. Collagen IV protein expression was normal in *Podxl* ^ΔEC^ lungs as assessed by immunoblot of homogenized lungs. Likewise, picrosirius red staining identified similar levels of collagen within the lung parenchymal tissue from *Podxl* ^F/F^ and *Podxl* ^ΔEC^ mice (data not shown).

Although picrosirius red is exceptional for identifying dense collagen, it cannot detect more subtle changes in small collagen fibers or alterations in areas where collagen is less abundant [42], [43]. Therefore, to further evaluate the distribution of collagen I matrix structural components, we used second harmonic generation (SHG) paired with multi-photon excitation fluorescence (MPEF) microscopy. Non-centrosymmetric collagen molecules (e.g., fibrillar collagen I) produce a specific second harmonic signal, while the auto-fluorescent signal indicates the presence of elastin, macrophages or red cells [42], [44]. SHG/MPEF microscopy allowed us to image both fibrillar collagen and elastin at high spatial resolution and specificity in three dimensions (3D) [45]. In contrast to healthy human alveolar tissue where the arrangement of fibrillar collagen and elastin complement each other [44], [45], healthy mouse alveolar tissue have less fibrillar collagen but, where present, it is consistently co-localized with elastin under non-pathologic conditions (**Fig. 5C** **, orange arrows**). Direct comparison of the distribution of collagen and elastin in *Podxl^F/F^* and *Podxl* ^ΔEC^ mice revealed a much higher frequency of elastin-free collagen fibrils in *Podxl* ^ΔEC^ alveolar tissue (**Fig. 5C** **, yellow arrows**). Using the co-localization scatter plots (**Fig. 5D**) we quantified the elastin-free-collagen and found a greater than 30% increase in *Podxl* ^ΔEC^ alveolar lung tissue (**Fig. 5E**). Taken together, the increased lung volume along with the increased amounts of elastin-free fibrillar collagen in alveolar space suggests that *Podxl* ^ΔEC^ mice have abnormal lung growth.

### *Podxl* ^ΔEC^ mice exhibit increased basal and inflammation-induced lung vascular permeability

**Figure 6:**
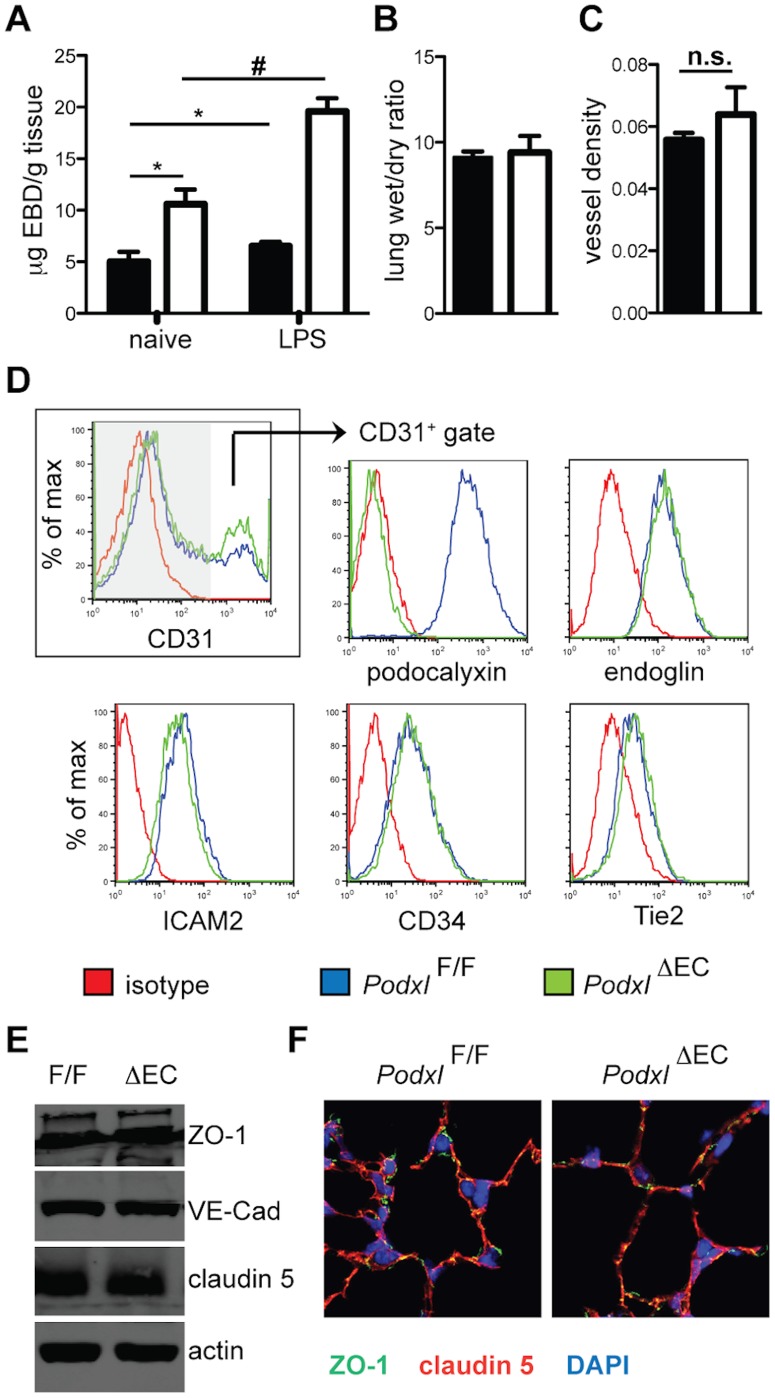
Podocalyxin deletion results in increased vascular permeability without altered endothelial cell frequency or immunophenotype. (**A**) Vascular permeability in lungs assessed by a modified Miles assay. Female mice were treated with PBS (naïve) or LPS (2 mg/kg) intra-tracheally 24 h before sacrifice *Podxl^F/F^* (black bars), and *Podxl* ^ΔEC^ (white bars) (n = 5–7 mice per genotype). One hour before sacrifice, mice received 20 mg/kg Evan's blue dye via the lateral tail vein. *Significantly different with P<0.05 by when compared to Podxl*^F/F^* naïve mice. #Significantly different (P<0.05) than LPS treated Podxl*^F/F^* mice by one-way ANOVA. (**B**) Lung edema presented as a ratio of wet/dry lung weight. Lungs were excised and weighed immediately for wet weight and subsequently dried at 60°C for 36 h and weighed again to determine the dry weight (n = 6 mice). (**C**) Vessel density was determined by the ratio of von Willebrand factor-positive (vWF^+^) staining to total lung tissue. Data represent 4 images per mouse and 6 mice per group. (**D**) Lung tissue displays normal expression of endothelial cell markers by flow cytometry. Shown are representative flow cytometry histograms. The frequency of CD31^+^ endothelial cells from each genotype is shown in the first profile. All subsequent profiles were gated on CD31^+^ cells in order to focus exclusively on marker expression by endothelia. (**E**) Expression of junctional proteins in lung. Lungs from *Podxl^F/F^* and *Podxl* ^ΔEC^ mice were homogenized in RIPA buffer and proteins were resolved on 8% SDS-PAGE gel followed by immunoblotting with the antibodies indicated. Actin was used as an internal loading control. The immunoblots shown are from one experiment and are representative examples of 4 independent mice/genotype. (**F**) Localization of junctional proteins in lung. Sections from inflated lungs of *Podxl^F/F^* and *Podxl* ^ΔEC^ mice were stained for ZO-1 (green), claudin-5 (red), and nuclei (DAPI, blue). In endothelial cells, ZO-1 and claudin-5 co-localize (yellow), and ZO-1 is also detected in epithelial cell junctions (green). The immunofluorescence micrographs are representative of 3 images per mouse and 3 mice per genotype.

Abnormal lung structure has previously been identified in mice exhibiting abnormal vascular integrity [46]. Therefore we performed a detailed assessment of vascular marker expression and vascular integrity on *Podxl* ^ΔEC^ lung endothelia. To evaluate the integrity of the vasculature, we performed Evan's blue dye (EBD) vascular leakage assays on naïve mice and mice treated with intra-tracheal LPS to induce acute lung inflammation. Naïve *Podxl* ^ΔEC^ mice exhibit a two-fold increase in permeability while *Podxl* ^ΔEC^ mice treated with LPS 24 hours before sacrifice exhibited an additional increase in permeability when compared to *Podxl^F/F^* mice (**Fig. 6A**). Although increased permeability is often associated with tissue edema, we observed no overt changes in the water weight of naïve *Podxl* ^ΔEC^ lungs (**Fig. 6B**) nor did we detect an obvious alteration in vascular density as determined by vWF staining (**Fig. 6C**). To further determine whether this enhanced permeability reflects a difference in the frequency or phenotype of endothelial cells, we evaluated collagenase-treated lung preparations by flow cytometry. This revealed a similar frequency of CD31^+^ endothelial cells in *Podxl^F/F^* and *Podxl* ^ΔEC^ lungs (**Fig. 6D**). Subsequent analysis of cells “gated” for a CD31^+^CD45^−^ phenotype revealed identical expression levels of CD34, Tie2, endoglin, and ICAM-2 and a selective loss of podocalyxin expression on *Podxl* ^ΔEC^ endothelia (**Fig. 6D**). Thus, the observed increased permeability occurs in the absence of major changes in endothelial cell frequency or expression of a range of lineage markers.

### Loss of podocalyxin alters endothelial cell-matrix interactions through modulation of integrin and matrix protein expression

Vascular permeability is regulated by two key factors; cell-cell contact mediated by endothelial junctional proteins and cell-matrix interactions mediated by integrins. To test the former, we examined expression levels and localization of the junctional proteins VE-cadherin, claudin 5, and ZO-1 in lung tissue (**Fig. 6E–F**). We did not observe any overt differences in expression of these proteins by immunoblot (**Fig. 6E**) nor did we observe a difference in their localization by immunofluorescence microscopy (**Fig. 6F**).

**Figure 7:**
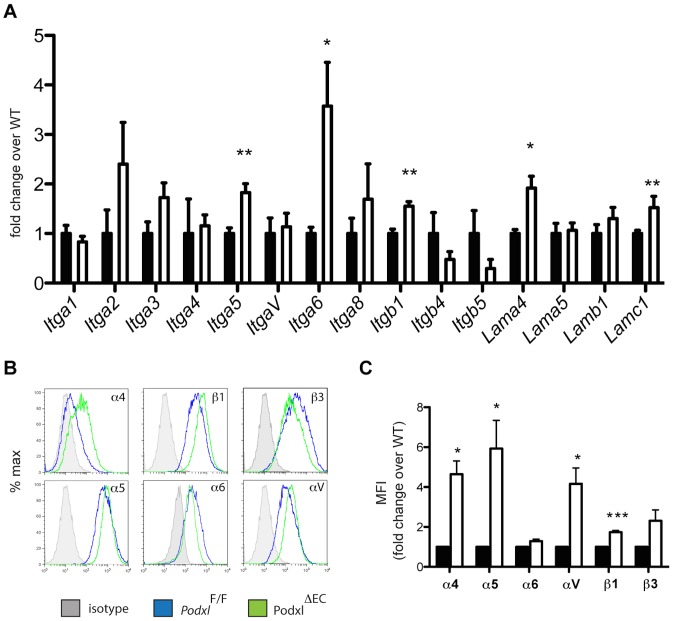
Podocalyxin deletion results in altered integrin and laminin expression in primary lung endothelial cells. (**A**) Integrins gene expression in *Podxl^F/F^* (black bars) and *Podxl* ^ΔEC^ (white bars) cultured lung mEC (results are the mean of 3 independent mEC primary cultures per genotype). (**B**) Cell surface expression of integrins on lung mECs isolated from *Podxl* ^F/F^ (blue line) and *Podxl* ^ΔEC^ (green line) mice. Shown are representative histograms from flow cytometric assays from one experiment. (**C**) Surface expression levels of integrins were determined by flow cytometry using the mean fluorescence intensity (MFI) of the integrin staining in primary mEC cultures. The mean change in the MFI of *Podxl* ^ΔEC^ mECs (white bars) compared to *Podxl* ^F/F^ mEC (black bars, normalized to 1) are from 4 independently derived mEC cultures. Error bars  =  SEM, *Significantly different with P<0.05 or ***significantly different with P<0.005 using one-sample *t* test with hypothetical value set to 1 (normalized control).

Cell adhesion to the extracellular matrix is largely determined by the expression and activation status of integrin-type adhesion molecules, which are broadly expressed by all cells in the lung. We therefore isolated primary mouse pulmonary endothelial cells (mEC) from individual *Podxl* ^F/F^ and *Podxl* ^ΔEC^ mice and evaluated the expression levels of individual integrin alpha and beta subunits encoding the proteins responsible for binding fibronectin, laminin and collagen. Primary mouse endothelia, in contrast to their human counterparts, are notoriously difficult to maintain *in vitro* when isolated from individual mice. However we were able to maintain comparable cultures of *Podxl* ^F/F^ and *Podxl* ^ΔEC^ endothelia for up to five passages. In primary lung mECs, we found a statistically significant increase in the expression levels of integrin α5 (*Itga5*), α6 (*Itga6*), and β1 (*Itgb1*) in *Podxl* ^ΔEC^ lung mEC when compared to *Podxl* ^F/F^ cells (**Fig. 7A**). At the protein level, we found that *Podxl* ^ΔEC^ mECs upregulate surface expression of α4, α5, and β1 integrin chains (**Fig. 7B&C**). However, α6 and β3 integrin expression were similar in *Podxl* ^ΔEC^ and WT primary lung ECs.

**Figure 8:**
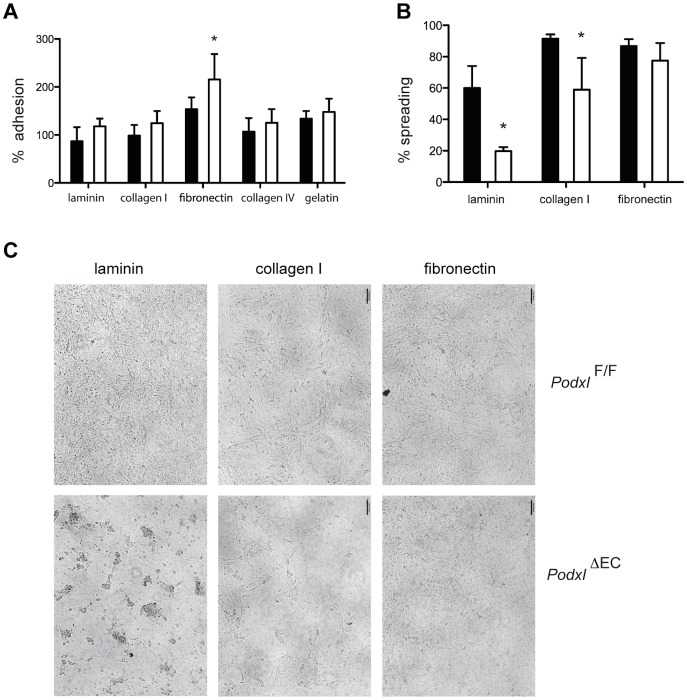
Podocalyxin expression on lung endothelial cells is required for efficient spreading on laminin and collagen I matrices. (**A**) Static adhesion of lung mECs isolated from *Podxl^F/F^* (black bars) and *Podxl* ^ΔEC^ (white bars) mice to surfaces coated with fibronectin, laminin, gelatin, collagen I, and collagen IV. Primary mEC were plated on matrix-coated plates for 90 min, washed and adhesion quantified by crystal violet absorbance. The average absorbance for the uncoated wells was normalized to 1 and the relative absorbance (proportional to adhesion) is shown. (**B**) Spreading of lung mECs isolated from *Podxl^F/F^* (black bars) and *Podxl* ^ΔEC^ (white bars) mice. Primary mECs were plated on matrix-coated transwells (0.4 µm pore size) and cultured for 48 h. The % monolayer coverage (spreading) was assessed by the threshold area of the cell monolayer via ImageJ on at least 3 independent cultures per genotype. The mean adhesion reported here was pooled from two independent experiments. (**C**) Representative bright field micrographs of spreading assay described in (E). Error bars  =  SEM, *Significantly different with P<0.05 by Student's *t* test or one-sample.

The alterations in integrin expression suggested a possible change in cell adhesion or spreading and therefore to test whether podocalyxin alters endothelial cell adhesion, lung mECs were allowed to adhere to tissue culture wells coated with fibronectin, laminin, collagen I, collagen IV or gelatin for 90 min. This time frame is sufficient to permit static adhesion, but does not permit extensive cell spreading. As expected by the upregulation of α4β1 *Podxl* ^ΔEC^ ECs exhibited a clear increase in static adhesion to fibronectin but not to laminin or collagen (type I or type IV) (**Fig. 8A**). We then repeated this assay in a fashion that would permit the evaluation of endothelial spreading. Lung mEC were plated on 0.4 µm pore transwell inserts coated with fibronectin, collagen or laminin, which provide an environment that is more typical of an *in vivo* setting for endothelial spreading. Control and podocalyxin-deficient lung mECs exhibit a similar ability to spread on fibronectin-coated inserts over 48 hours (**Fig. 8B&C**). Strikingly, *Podxl* ^ΔEC^ lung mEC exhibited a severe impairment in their ability to spread on laminin-coated inserts in comparison to control lung mEC. Although *Podxl* ^ΔEC^ lung mEC were observed to make initial contact with the substrate, they failed to flatten and elongate (**Fig. 8C**). Instead, these cells formed clumps that remained attached to the insert surface over the 48-hour course of the assay. Plating *Podxl* ^ΔEC^ lung mEC on collagen I yielded an intermediate phenotype; the cells displayed the ability to spread on this matrix, but not as readily as control lung mEC (**Fig. 8B&C**). The lung endothelial basement membrane is comprised primarily of laminin-8 and laminin-10, which are composed of chainsα4β1γ1 or α5β5γ1 respectively [47]. Importantly, laminin α4and γ1 transcripts were both upregulated in *Podxl* ^ΔEC^ mEC suggesting an increase in laminin-8 synthesis (**Fig. 7A**). In summary, our data suggest loss of podocalyxin from lung vascular endothelia leads to a highly selective defect in laminin-dependent adhesion that results in increases vascular permeability/leakage and vascular-induced remodeling of the lung parenchyma.

## Discussion

Although luminal expression of podocalyxin in endothelial cells was documented over 20 years ago [12] its function in the vasculature is unknown. Previously, we used gene targeting strategies to show that podocalyxin plays an essential role in kidney podocyte morphogenesis and that its ablation leads to anuria, hypertension and perinatal death [18]. To date, this remains the only mucin knockout mouse to show such a profound and lethal phenotype. Despite the utility of this mouse model in revealing a critical function for podocalyxin on podocytes, several questions have remained, including the degree of vascular contribution to the severity of this phenotype. Here we have addressed this question by generating a vascular-specific lesion in the podocalyxin gene.

We focused our attention on the lung due to the highly efficient deletion of *Podxl* in the lung endothelia, and because recent work has highlighted the importance of endothelial integrity in lung development, function, and regeneration [5], [6], [9]. The defects we observe are highly specific. Like *Podxl^-/-^* mice, *Podxl* ^ΔEC^ mice exhibit normal lung architecture at birth, arguing against an essential role in branching morphogenesis [18]. Alveolarization is often affected by the disruption of endothelial cell growth or signaling as is the case in mice deficient for CD31, eNOS and SDF-1/CXCL12 [46], [48], [49]. Similarly, disruption of VEGF or its receptors is sufficient to cause endothelial cell dysfunction and apoptosis, which leads to defects in alveolarization and lung maintenance [50]–[52]. In contrast, *Podxl* ^ΔEC^ mice exhibit normal post-natal alveolarization but then show a clear vascular-driven abnormal lung growth. This coincides with a decrease in elastin fibers (at the protein level), the development of elastin-free fibrillar collagen and functional changes to the lungs, namely increased resistance in both the airways and chest as a whole, which taken together, are all indicative of early stages of emphysema [53]–[56]. While increased collagen levels may not be commonly associated with emphysema, in both samples from patients as well as in elastase-induced models of emphysema, thickened collagen fibrils can be found in areas of lung where the elastin has been degraded [54]. More recently, structure-function studies identified these same areas of fibrillar collagen devoid of elastin to be prone to tissue failure, leading to decreased tissue density [57]. Taken together, our data suggest loss of podocalyxin is sufficient to induce a post-natal defect in lung maintenance or regeneration, reminiscent of a pulmonary “emphysema-like” phenotype. This reinforces a role for the adult endothelium in regulating airway epithelial architecture and function.

In epithelial cells, podocalyxin expression controls cell adhesion and spreading by defining the apical domain, facilitating the sorting of integrins to the proper localization and regulating the contribution of the actin cytoskeleton [10], [22], [58]. In addition, we have shown previously that overexpression of podocalyxin in epithelial cells (OVCAR-3 cells, an ovarian carcinoma cell line) is sufficient to decrease adhesion to fibronectin and to moderately down regulate surface expression of β1 integrin, a key component of the fibronectin receptor [59]. In the present study, we find that, correspondingly, loss of podocalyxin is sufficient to increase β1 integrin mRNA and surface expression, as well as increase the static adhesion of mEC to fibronectin. Thus, there is accumulating evidence to suggest an inverse relationship between podocalyxin and integrin expression.

A novel observation in the current study was the altered behaviour of *Podxl* ^ΔEC^ mEC on laminin and collagen. Although *Podxl* ^ΔEC^ mEC show a normal ability to make initial contact with these matrices, they exhibit a striking inability to spread effectively on laminin and, to a lesser extent on collagen. While the cause of this matrix-specific spreading defect is currently unknown, this phenotype is reminiscent of the inability of podocytes in *Podxl^-/-^* mice to spread on the laminin-rich glomerular basement membrane [18]. Likewise, glomerular endothelial cells lacking podocalyxin are thickened and fail to form appropriate fenestrae [18]. Thus, there is precedence to suggest that podocalyxin regulates the morphogenesis of both epithelial and endothelial cells bound to laminin-rich basement membranes. In addition, we have demonstrated that in the lung *Podxl* ^ΔEC^ mice upregulate expression of laminin-8 subunits. Laminins-8 and -10 constitute the primary basement membrane for endothelial cells in the lung, and laminin-8, due to a lack of globular domains on its short arms, is thought to provide a provisional basement membrane scaffold with collagen IV prior to the assembly of a mature basement membrane composed of laminin-10 (reviewed in [60]). Therefore, in the absence of podocalyxin, the increased production of laminin 8 may suffice to allow normal vessel development but be insufficient to maintain vessel integrity leading to the observed increase in permeability in podocalyxin deficient mice. In vitro, this could also lead to an inability to spread on laminin-coated surfaces due to overproduction of endogenous laminin-8 by knockout endothelial cells (work in progress).

Given that lung endothelial cells express CD34 (and perhaps endoglycan) podocalyxin's closest relatives, it is conceivable that one or both of these other family members may partially compensate for the absence of podocalyxin in *Podxl* ^ΔEC^ mice. The generation of compound knockout mice for additional CD34-related mucin genes offers an opportunity to address this hypothesis (in progress).
