# Correction: Podocalyxin Regulates Murine Lung Vascular Permeability by Altering Endothelial Cell Adhesion

**DOI:** 10.1371/journal.pone.0116613

**Published:** 2014-12-23

**Authors:** The PLOS ONE Staff

The fourth author’s name is spelled incorrectly. The correct name is: Alex Lu. The correct citation is: Debruin EJ, Hughes MR, Sina C, Lu A, Cait J, et al. (2014) Podocalyxin Regulates Murine Lung Vascular Permeability by Altering Endothelial Cell Adhesion. PLoS ONE 9(10): e108881. doi:10.1371/journal.pone.0108881
